# Supplementary figures and images for: Access to HIV prevention, treatment, and care services during COVID-19 by men who have sex with men in Zimbabwe, An interpretive phenomenological analysis study
Source: PLoS One. 2023 Apr 14;18(4):e0281799. doi: 10.1371/journal.pone.0281799 (PMC10104336; doi:10.1371/journal.pone.0281799)

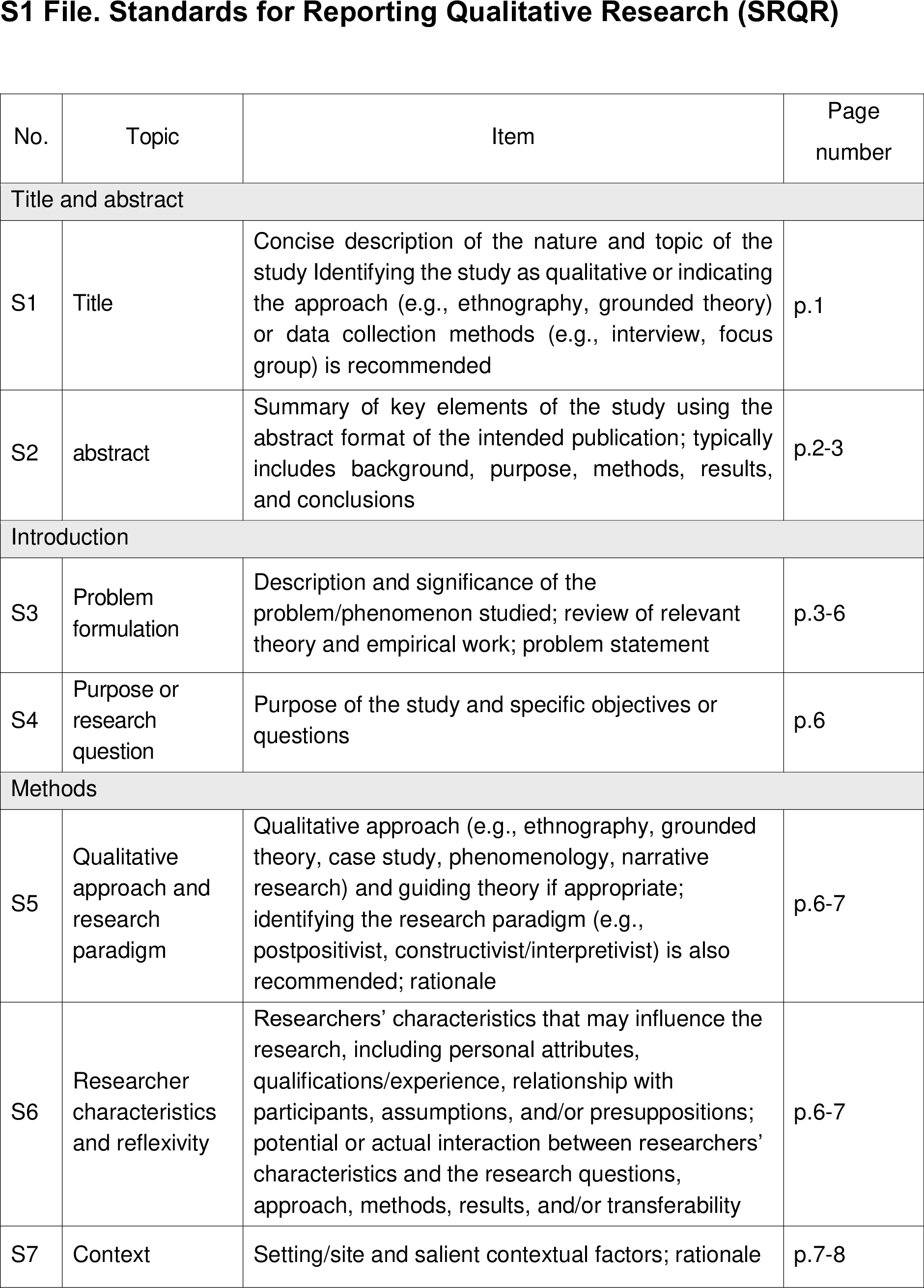

Supplement: S1 File — (ZIP) [file pone.0281799.s001.zip › S1 File. Standards for Reporting Qualitative Research p1.tif]

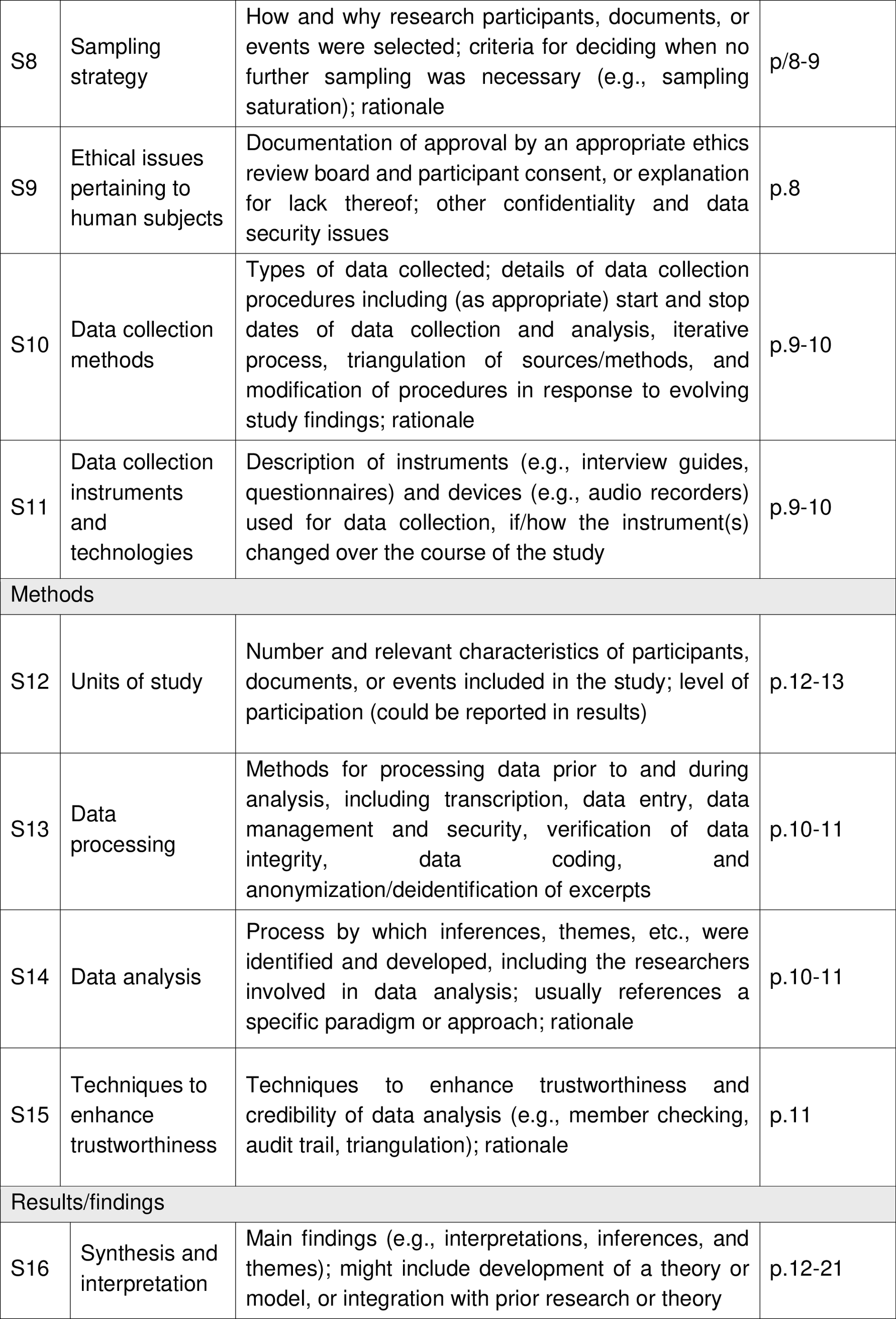

Supplement: S1 File — (ZIP) [file pone.0281799.s001.zip › S1 File. Standards for Reporting Qualitative Research p2.tif]

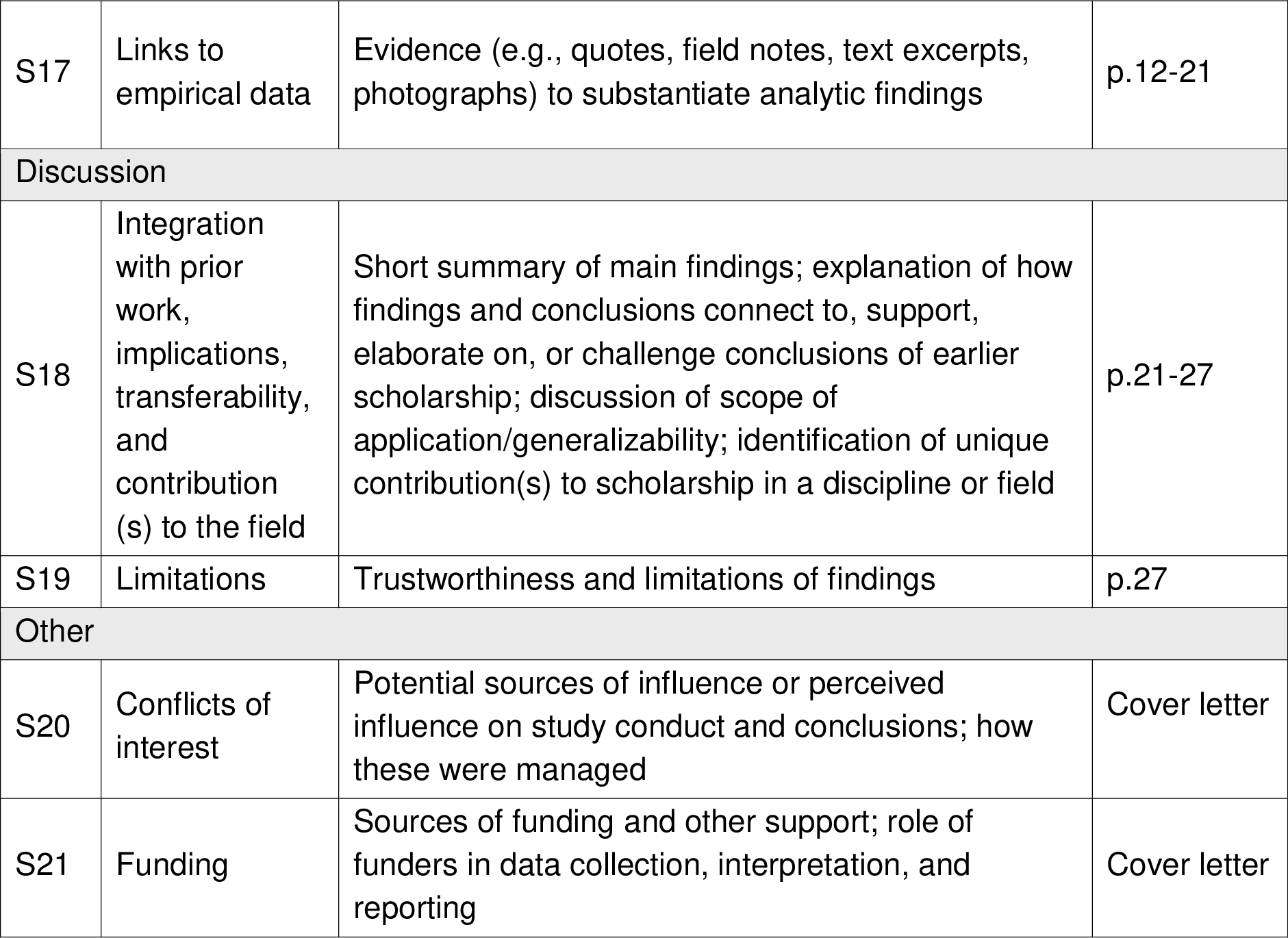

Supplement: S1 File — (ZIP) [file pone.0281799.s001.zip › S1 File. Standards for Reporting Qualitative Research p3.tif]

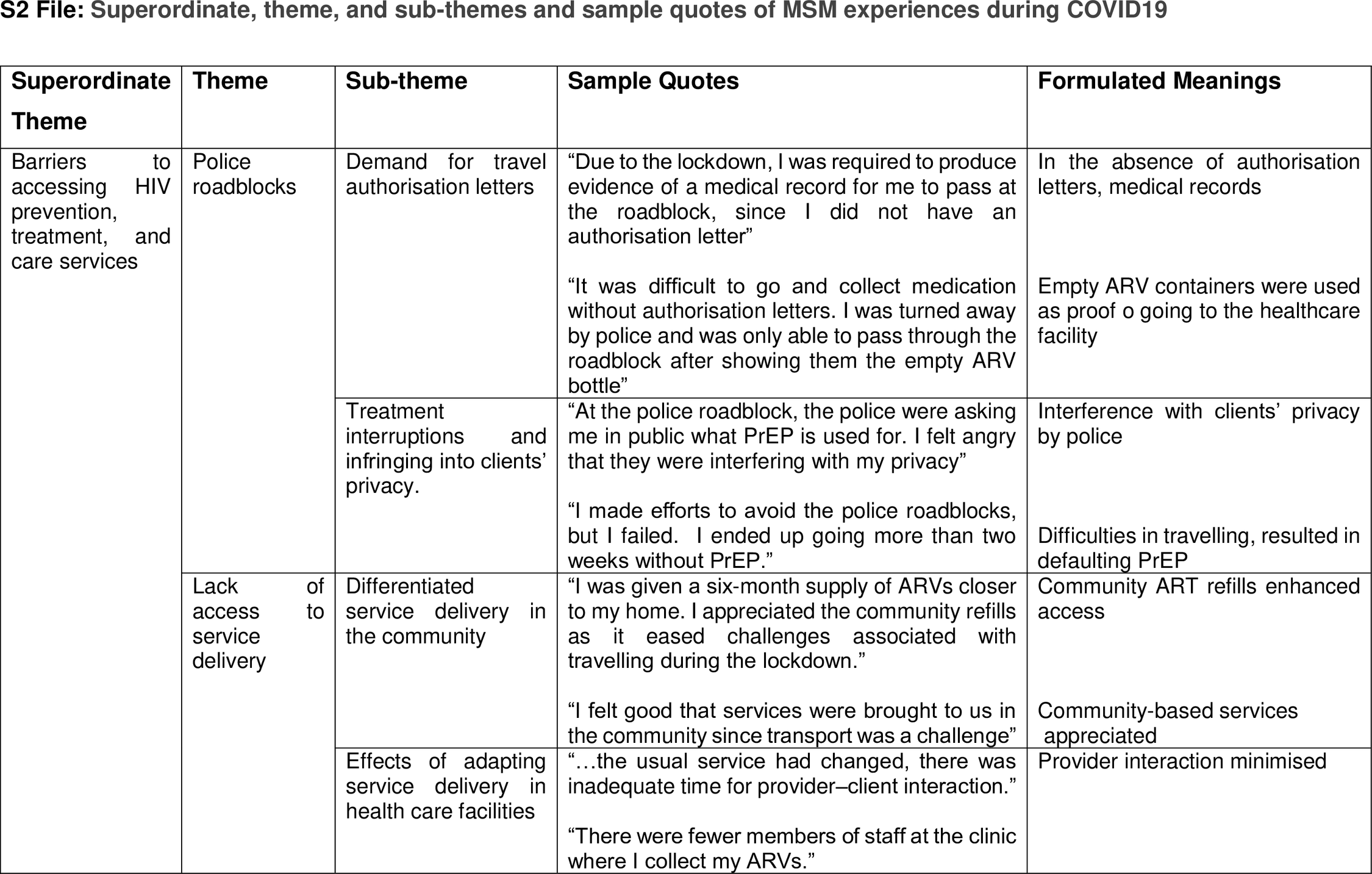

Supplement: S2 File — (TIF) [file pone.0281799.s002.tif]

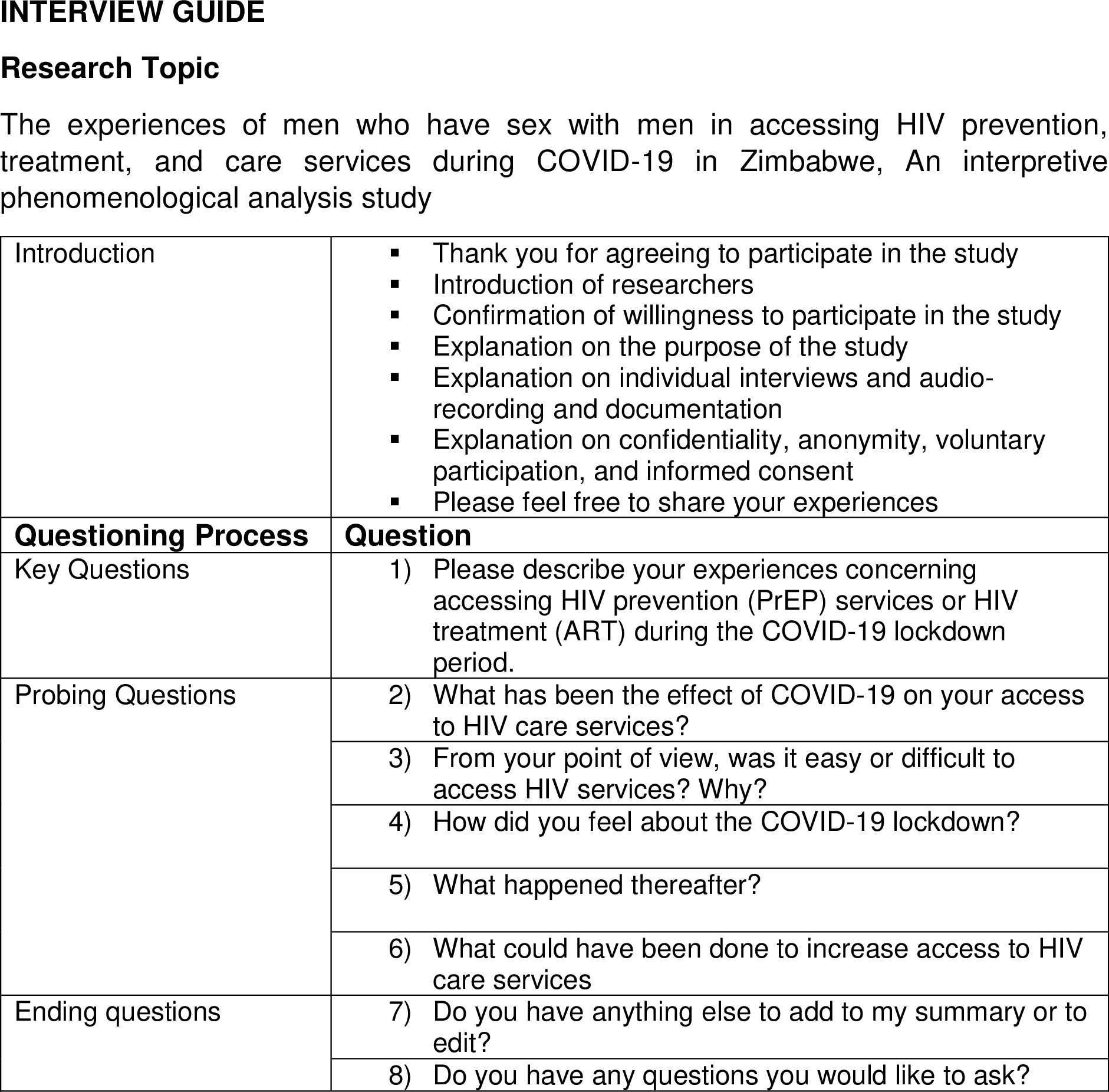

Supplement: S3 File — (TIF) [file pone.0281799.s003.tif]

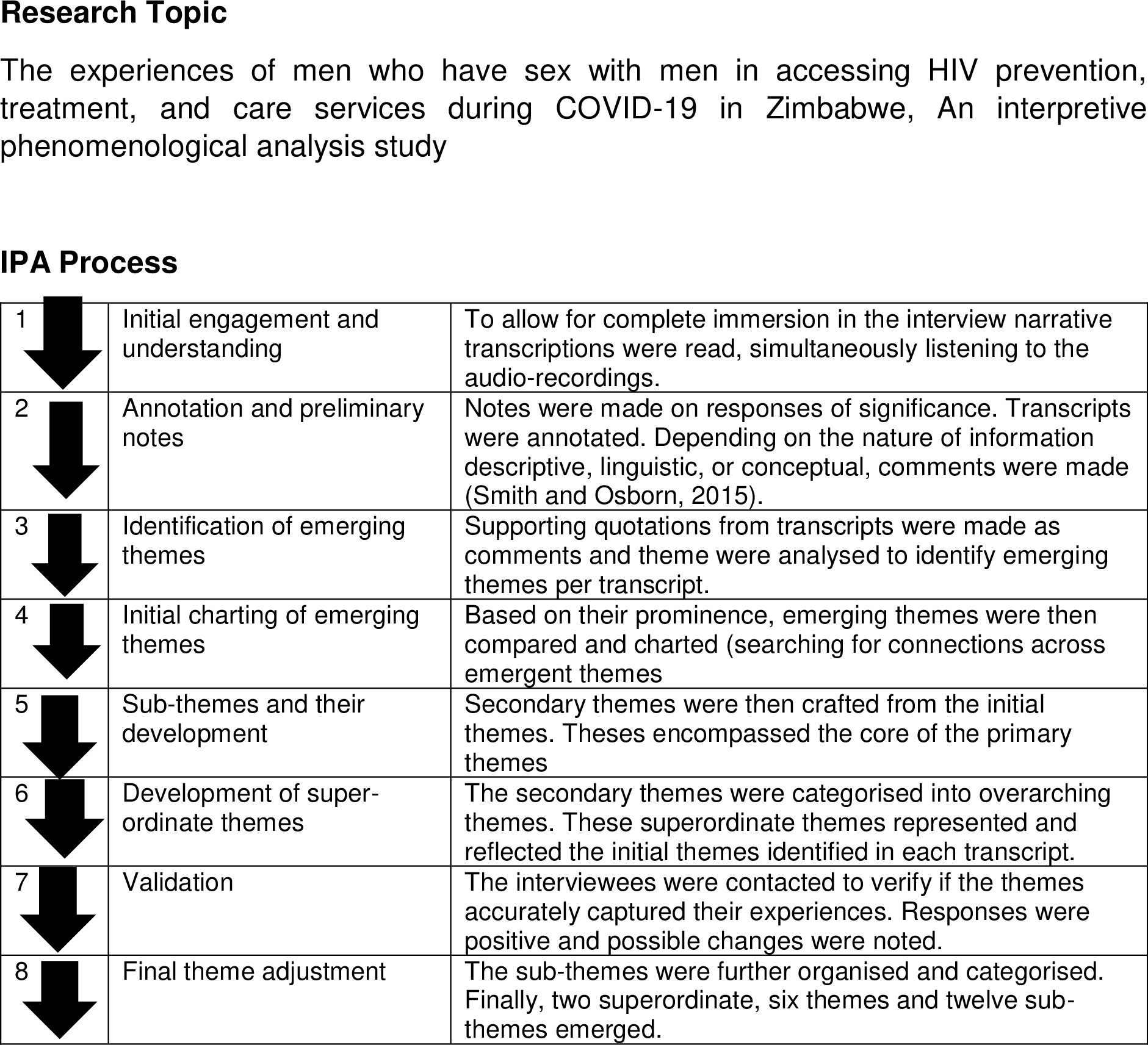

Supplement: S4 File — (TIF) [file pone.0281799.s004.tif]
