# Supplementary material for: Access to HIV prevention, treatment, and care services during COVID-19 by men who have sex with men in Zimbabwe, An interpretive phenomenological analysis study
Source: PLoS One. 2023 Apr 14;18(4):e0281799. doi: 10.1371/journal.pone.0281799 (PMC10104336; doi:10.1371/journal.pone.0281799)
Supplement: Attachment — Submitted filename: RESPONSE TO REVIEWERS COMMENTS.pdf [file pone.0281799.s007.pdf]

RESPONSE TO REVIEWERS' COMMENTS

No	Editor's comments	Response
1	The most substantial issue is Table 1 on page 14. Why are there two columns differentiating between sexual orientation (all listed as gay) and sexuality (all listed as homosexual)? This does not make conceptual or practical sense for two reasons. Firstly, 'homosexual' is generally avoided, due to its history as an overly medicalised term, and is now outdated. Secondly, there is no difference between 'gay' and 'homosexual' - what do the authors mean to imply here?	The table has been corrected and re-aligned
2	Related to the above issue is whether this is indeed a study of MSM at all, or a study on gay men in Zimbabwe? There are conceptual differences. The term "MSM" and "key populations" belongs to public health discourse and do not necessarily account for the actual identity of the people it homogenises under the label "MSM". A person listed as MSM might not be gay; they may be heterosexual or bisexual, etc. In your study, ALL the participants were listed as "gay" - how was this information given? Please provide the manner in which the demographic variables were obtained so that it is clear whether the participants were given pre-determined options for their 'sexual orientation' and 'sexuality' or whether there were opportunities for them to self-identify differently. If indeed all the participants IDENTIFIED as gay, then why was the study framed as a study of MSM and not as a study of gay men? Do you have evidence that the participants themselves prefer the term MSM? Where they asked?	The table has been corrected and re-aligned. Recruitment of the participant was according to the set inclusion criteria. The inclusion criteria for participating in the study were as follows: self-identifying as men who have sex with other men, aged 18 years or older and who receive ART or pre-exposure prophylaxis (PrEP) from any of the healthcare facilities in Bulawayo.
3	A few grammar errors, e.g. line 27, line 82	Grammatical errors corrected
4	The uncritical use of the phrase "hard to reach" on line 138 (there is a lot of critique around this term)	The term "hard to reach" was replaced by the term "difficult to access"
5	Remove "best opportunity" on line 146 as many methods can achieve your research goals.	The term "best opportunity" was removed, and the sentence was rephrased.
6	Line 153 you say "the study was performed" according to SRQR; however, the SRQR is not meant to dictate how to perform the actual study. It guides the reporting of the subsequent manuscript.	The sentence was rephrased

7	The SRQR S.6 requires you to comment on Researcher Characteristics. There is almost no reflexivity on the characteristics or positionality of the authors, in relation the participants. More detail is needed here: who are you, why did you personally want to research this area, what previous engagements have you had with similar participants, are any of your members of these communities? etc.	Researcher characteristics highlighted
8	Line 162 - what were the incentives to participate in this study? It is unclear why people chose to participate in a study that is potentially stigmatising to their identity/	Participation in the study was based on willingness after getting information on what the study entailed
9	"KP friendliness" - you have used the acronym "KP" before.	The term “KP” was written in full
10	You note that participants were selected "by virtue of their capacity to provide richly textured information". How did you know beforehand whether or not a potential participant would be able to provide "richly textured information"?	The paragraph was rephrased.
12	On Table 1, an international audience may not know what O-Level and A-Level are.	The table has been corrected and re-aligned. O-Level was replaced by ‘secondary school’ and A-Level by ‘High School’
13	On Table 1, under employment status, is there a difference between Self-Employed and 'Male Sex Worker'? Would a Male Sex Worker not also be self-employed in some instances?	The table has been corrected and re-aligned
