# Supplementary material for: Access to HIV prevention, treatment, and care services during COVID-19 by men who have sex with men in Zimbabwe, An interpretive phenomenological analysis study
Source: PLoS One. 2023 Apr 14;18(4):e0281799. doi: 10.1371/journal.pone.0281799 (PMC10104336; doi:10.1371/journal.pone.0281799)
Supplement: Attachment — Submitted filename: RESPONSE TO REVIEWERS COMMENTS 14012023.pdf [file pone.0281799.s008.pdf]

RESPONSE TO REVIEWERS' COMMENTS

No	Editor's comments	Response
1	Avoid phrases like "the MSM" in the abstract - this can be objectifying by using "the".	The phrase "like MSM" was removed from the abstract
2	Some grammatical issues are still in the manuscript e.g lines 149-150; 167-168; 186-187; 239; Table 1 (note: I am using line numbers in the Tracked Changed version of the manuscript).	Grammatical areas were addressed as shown by tracked changes
3	Phrases like "hard-to-reach population" are still used in the manuscript (e.g. line 190), despite criticisms in the previous review and depite the authors claiming that "The term " 'hard to reach' was replaced by the term "difficult to access".	This was corrected. The term "hard to reach" was replaced by the term "difficult to access"
4	On line 290 you note that the participants "self-identified as MSM" (presumably as per the inclusion criteria), however, further to this, did they also self-identify as "homosexual" or would they have preferred to be described as "gay"?<br>I ask this because there are still references to "the gay community" (e.g. line 171). You need to add a footnote or in-text clarification about whether your participants self-identify as "gay" and/or "homosexual" and/or "MSM" (and/or how these categories of identity overlap, if at all). This is important, in order to be ethical and respectful of participants' identities. This speaks to the politics of representation in qualitative research.	This section has been corrected and aligned accordingly. More information has been added under Biographic information section to enhance clarity.
