# Supplementary material for: Access to HIV prevention, treatment, and care services during COVID-19 by men who have sex with men in Zimbabwe, An interpretive phenomenological analysis study
Source: PLoS One. 2023 Apr 14;18(4):e0281799. doi: 10.1371/journal.pone.0281799 (PMC10104336; doi:10.1371/journal.pone.0281799)
Supplement: Attachment — Submitted filename: Reviewer.docx [file pone.0281799.s005.docx]

This is a well written manuscript detailing The experiences of men who have sex with men in accessing HIV prevention, treatment, and care services during COVID-19 in Zimbabwe. A few comments

1. Line 179-181, the statements ‘Therefore, probes and prompts were used to elicit more 180 detailed information from participants [19]. Therefore, probes and prompts were used to elicit more detailed information from participants [15,19]’

are repetitive.

2. Table1, duration on what? It would be better to clearly state on the subtitle
3. Line 255, did you intended to say …. Had had…., cross check
