# Supplementary material for: Access to HIV prevention, treatment, and care services during COVID-19 by men who have sex with men in Zimbabwe, An interpretive phenomenological analysis study
Source: PLoS One. 2023 Apr 14;18(4):e0281799. doi: 10.1371/journal.pone.0281799 (PMC10104336; doi:10.1371/journal.pone.0281799)
Supplement: Attachment — Submitted filename: RESPONSE TO REVIEWERS COMMENTS 200522.docx [file pone.0281799.s006.docx]

**RESPONSE TO REVIEWERS’ COMMENTS**

| **No** | **Editor’s comments** | **Response** |
| --- | --- | --- |
| 1 | Please ensure that your manuscript meets PLOS ONE's style requirements, including those for file naming. | Manuscript has been aligned according to PLOS ONE Journal requirements  Files have also been named according to journal expectations and requirements |
| 2 | When reporting the results of qualitative research, we suggest consulting the COREQ guidelines or other relevant checklists listed by the Equator Network, such as the SRQR, to ensure complete reporting.   Moreover, please provide the interview guide used as a Supplementary File. | Standards for Reporting Qualitative Research (SRQR)  Interview guide is provided, submitted as a supplementary file |
| 3 | In your Data Availability statement, you have not specified where the minimal data set underlying the results described in your manuscript can be found. PLOS defines a study's minimal data set as the underlying data used to reach the conclusions drawn in the manuscript and any additional data required to replicate the reported study findings in their entirety. | The minimal data set are available are reported under the Supplemental Information (S2 File showing superordinate themes, themes, sub-themes and related excerpts) |
| 4 | We note you have included a table to which you do not refer in the text of your manuscript. Please ensure that you refer to Table 1 in your text; if accepted, production will need this reference to link the reader to the Table. | In the manuscript table 1 has been referred to. |

**Reviewer 1**

| **No** | **Reviewer comments** | **Response to reviewers’ comments** |
| --- | --- | --- |
| 1 | Line 179-181, the statements ‘Therefore, probes and prompts were used to elicit more 180 detailed information from participants [19]. Therefore, probes and prompts were used to elicit more detailed information from participants [15,19]’  are repetitive. | Repetition was eliminated |
| 2 | Table1, duration on what? It would be better to clearly state on the subtitle | Information added to enhance clarity-Duration on PrEP/ART |
| 3 | Line 255, did you intended to say …. Had had…., cross check | Cross checked. The authors intended to use ‘had had’ |

**Reviewer 2**

| **No** | **Section** | **Reviewer comments** | **Response to reviewers’ comments** | |
| --- | --- | --- | --- | --- |
| 1 | **Title** | The title should mention that this is a qualitative analysis | The term ‘An interpretive phenomenological analysis study’ was added at the end of the original title | |
| 2 | **Abstract**  Methods Section | In the methods section of the abstract, the authors should mention the data collection methods (ie we conducted in depth interviews with …) before the data analysis methods (IPA design) | An interpretative phenomenological analysis (IPA) design was used; hence it was mentioned before data collection. Then for data analysis ‘IPA framework for data analysis’ as described by Smith and Osborn (2015) was used. | |
|  | Results | “Results showed several barriers to accessing HIV services by MSM that warranted adjustments to service delivery.”  This sentence should be more specific as it does not provide substantial information | More information was added to enhance clarity | |
|  |  | “The study also found that COVID-19 and related restrictive measures had psychosocial and economic effects that included loss of income, intimate partner violence and psychological effects.” This sentence could also be more specific.’ | More information was added to make the sentence more specific. | |
|  | Conclusion | Conclusions suggest community-based HIV services, but this is not mentioned in the results. | The conclusion and the results have been synchronised by adding the information on community-based HIV services in the results section | |
| **Main document** | | | | |
| 3 | **Introduction** | “These measures, though well-meant, have had a negative impact on HIV services, resulting in challenges and risks to the infected and affected groups” This sentence makes it seems as though COVID restrictions were misguided although “well meant”. Do the authors think lockdowns were not necessary to prevent deaths due to COVID? If not, I suggest removing “tough” and “well meant” when talking about COVID protocols. The rest of this sentence is vague and provides no information about how COVID has affected key populations. Finally, I would avoid using “the infected” to talk about people with HIV. | The terms ‘though well-meant; tough; infected’ have been dropped as recommended | |
|  |  | “The Joint United Nations Programme on HIV/AIDS (UNAIDS) [2] indicates that key populations are particularly vulnerable to HIV service interruptions.”  Why is this the case? What kind of disruptions have occurred? | The introduction has been restructured to address the mentioned concern.  Concerning treatment/service disruptions that occurred, examples have been cited on closure facilities in Kenya and Zimbabwe (see line 63 to 67) | |
|  |  | “The emergence of COVID-19, together with the associated national lockdowns and restricted movement, has brought diverse challenges that further aggravate the situation for key populations.”  This sentence is vague and provides no information. What are the “diverse challenges”? | Information has been added to elaborate on the diverse challenges | |
|  |  | “According to UNAIDS [2], the COVID-19  84 pandemic has affected everyone, inclusive of key populations, who are at a  85 higher risk of acquiring HIV.”  This sentence is vague | The vagueness has been addressed by re-doing the introduction | |
|  |  | “The Joint United Nations Programme on HIV/AIDS indicates that the gains that have been made in implementing HIV programmes and increasing access to HIV services for key populations are at risk of being reversed as a result of disruptions caused by COVID-19”  This sentence is vague. | Introduction has been re-structured, and the sentence removed. | |
|  |  | “To curb the spread of COVID-19, the World Health Organization (WHO) called for adjustments in HIV care service delivery [1].”  What adjustments? This sentence is vague. | To enhance clarity, the mentioned adjustments were added | |
|  |  | The introduction needs to be completely re-written. It provides no information about the impact of COVID-19 in Zimbabwe, the characteristics and health seeking behavior of MSM in Zimbabwe, previous research on MSM’s engagement and experience with HIV services, gaps in the research and the need for the present study. There is insufficient description of the objectives of the current study (“other than explore the experience…”) and the introduction is too long. | The introduction has been re-written. The objectives and rationale were re-visited. | |
| 4 | **Methods** | Design—the IPA analysis should go in the analysis and not design section | According to Creswell and Creswell (2017) and Alase (2017), the interpretative phenomenological analysis (IPA) design-is a design hence the authors put it under the design section. For analysis purposes, the IPA Analysis framework (as outlined by Smith and Osborn, 2015) was utilised. | |
|  | Ethical considerations | This section should be greatly shortened | The section on ‘Ethical Considerations’ was restructured and shortened | |
|  | Sampling | “Purposive sampling facilitated selection of participants by virtue of their capacity to provide richly textured information, relevant to the phenomenon under study.” This sentence in unclear.  The sample size is very small (14 people). This should be mentioned in the limitations. | The sentence was rephrased to avoid ambiguity.  Methodological authorities have been cited to justify the sample size used. The sentence below was added to enhance clarity on sample size.  “The sample size of 14 is justifiable in a phenomenological study as supported by Polit and Beck (2018) who reported a sample of ten or more as adequate. This is corroborated by DeJonckheere and Vaughn (2019) who posit that a sample of six to twenty is suitable for a phenomenological study”. | |
|  | Data collection | It would be helpful to include the interview guide as an appendix  Interview guide: “From your point of view, what are the factors that facilitated or impeded your access to HIV prevention, treatment and care services?” This seems like a hard question | Interview guide was included.  The question was rephrased to read: ‘From your point of view, was it easy or difficult to access HIV services? Why?’ | |
|  | Data analysis | “Interpretative phenomenological analysis (IPA), an approach which aims to provide detailed examinations of personal lived experience [15].” This sentence is repetitive and is mentioned several times throughout the paper. | Repetition has been eliminated. | |
|  | Measures of trustworthiness | The purpose of this section is unclear. | The section on measures of trustworthiness was restructured to reflect validation of results | |
|  | Reflective diary | “A reflective diary was kept ensuring neutrality and objectivity.” The meaning of this is unclear | The sentence was removed to enhance clarity | |
| 5 | **Results** | Table one should be a summary of demographics and not a list of every single participant | The reviewer’s recommendation is appreciated, however after making a summary of the table as proposed, I felt it would not be easy for the readers to follow the summarised table. Therefore, would prefer to leave it as is. | |
|  |  | Loss of income and change in lifestyle seems like they could be combined into one theme Are there quotes to illustrate the difference? | The change of lifestyle is a sub-theme of the theme ‘loss of income’.  There are quotes illustrating changes in lifestyle arising from the loss of income. | |
| 6 | **Limitations** | One limitation to note in the “limitations” section is that it’s not possible to assess generalizability when using snowball sampling. | The limitation of snowballing sampling has been acknowledged | |
| 7 | **Conclusion** | The authors state “Moreover, law enforcement agents should be trained in more humane methods of policing” Can you provide examples of how this can be done or prior interventions in the literature that have been successful | | More information was added to added to enhance clarity |
|  |  | Can the authors suggest potential interventions to address the barriers and consequences of COVID observed in this study? Eg, loss of employment/income, IPV, depression/anxiety due to COVID and the lockdowns | Potential interventions were recommended. | |
|  |  | Are the negative experiences noted here specific to MSM or are they more generally experienced in the community due to COVID? How are the experiences of MSM distinct from the general community? | Some of the experiences may be generalised but the impact on a group that is already marginalised and stigmatised would definitely be different. Specific examples that relate to the MSM would include stigmatisation following forced disclosure on that the participants were taking pre-exposure prophylaxis and assumed to be a sex worker. | |
